# Achievement Goal Orientations and Adolescents’ Subjective Well-Being in School: The Mediating Roles of Academic Social Comparison Directions

**DOI:** 10.3389/fpsyg.2017.00037

**Published:** 2017-01-31

**Authors:** Lili Tian, Tingting Yu, E. Scott Huebner

**Affiliations:** ^1^School of Psychology, Guangdong Key Laboratory of Mental Health and Cognitive Science, Center for Studies of Psychological Application, South China Normal UniversityGuangzhou, China; ^2^Department of Psychology, University of South CarolinaColumbia, SC, USA

**Keywords:** achievement goal orientations, academic social comparison directions, subjective well-being in school, mediation, adolescents

## Abstract

The purpose of this study was to examine the multiple mediational roles of academic social comparison directions (upward academic social comparison and downward academic social comparison) on the relationships between achievement goal orientations (i.e., mastery goals, performance-approach goals, and performance-avoidance goals) and subjective well-being (SWB) in school (school satisfaction, school affect) in adolescent students in China. A total of 883 Chinese adolescent students (430 males; Mean age = 12.99) completed a multi-measure questionnaire. Structural equation modeling was used to examine the hypotheses. Results indicated that (1) mastery goal orientations and performance-approach goal orientations both showed a statistically significant, positive correlation with SWB in school whereas performance-avoidance goal orientations showed a statistically significant, negative correlation with SWB in school among adolescents; (2) upward academic social comparisons mediated the relation between the three types of achievement goal orientations (i.e., mastery goals, performance-approach goals, and performance-avoidance goals) and SWB in school; (3) downward academic social comparisons mediated the relation between mastery goal orientations and SWB in school as well as the relation between performance-avoidance goal orientations and SWB in school. The findings suggest possible important cultural differences in the antecedents of SWB in school in adolescent students in China compared to adolescent students in Western nations.

## Introduction

For adolescents, schools represent one of their major life domains (Huebner, 1994). Adolescents devote substantial amounts of time and effort to school experiences, and their school experiences play an important role in facilitating or inhibiting successful lifelong development (Schaps and Solomon, 2003). For many years, educational leaders and professionals in China and many other nations have paid much greater attention to the academic achievement of students than to students’ subjective well-being (SWB) in school (Dello-Iacovo, 2009). Nevertheless, among others, Noddings (2003) has argued persuasively that “Happiness and education are, properly, intimately connected. Happiness should be an aim of education, and a good education should contribute significantly to personal and collective happiness” (p. 1). From such a perspective, a good education should be concerned about students’ academic learning *and* SWB in school. In recent years, it is gratifying that some scholars have attached importance to SWB in school (e.g., Konu et al., 2015; Tian et al., 2015b; Persson et al., 2016), and evidence is accumulating that indeed SWB and academic achievement and behavior in school are reciprocally related (Suldo et al., 2011; Lyons et al., 2014; Ng et al., 2015).

Given that scholastic learning and social interactions represent two main activities in adolescents’ school lives, it is not surprising that adolescents’ self perceptions in academic and social domains should exert a significant impact on SWB in school. According to achievement goal theory (Midgley, 2002), achievement goals can be subdivided into different orientations. Regarding the area of school achievement in particular, achievement goal orientations refer to students’ general tendencies for approaching, engaging in, and evaluating their academic progress and performance in achievement contexts. Achievement goal orientations address the question of “why” individuals attempt to accomplish certain achievement outcomes (Elliot, 1997; Pintrich, 2000). Achievement goal orientations are an important line of research in the field of achievement motivation. Students’ well-being is associated with the goal orientations they pursue in achievement situations. Specifically, goal orientations related to achieving self-improvement and growth are associated significantly with adaptive socio-emotional functioning and positive overall adjustment, whereas goal orientations related to demonstrating higher levels of competence compared to others are associated significantly with socio-emotional vulnerability and poor overall adjustment (e.g., Dykman, 1998; Daniels et al., 2008). Therefore, we explored the specific relations between different achievement goal orientations and SWB in school, including possible psychological mechanisms that might account for the observed relations.

The influence of people’s motives and goals on SWB may depend on the environmental context (see Diener et al., 1999, for a review). An important component of context is the culture in which the individual is immersed. East Asian cultural members possess a highly context-sensitive self, which is defined as variability of the phenomenal self across contexts (Kashima et al., 2004; Suh, 2007). Specifically, China has been influenced by a Confucian worldview that recognizes the self to be most meaningful in relation to other selves. In a sense, each self is a necessary element for other selves, thus interpersonal relationships reflect the critical building blocks of Chinese life (Heine, 2001). Compared with Westerners, East Asians are less likely to refer to their inner thoughts and feelings when evaluating themselves; they are inclined to refer to interactions with others in context (Suh, 2007). Such trends generalize to judgments of SWB. East Asians are less likely to take their inner emotions into account when they make life-satisfaction judgments (Suh et al., 1998). On the contrary, socially nuanced cues are given more weight, such as social comparison information (e.g., Oishi and Diener, 2001; White and Lehman, 2005). Therefore, comparison theory may help explain the psychological mechanisms that mediate associations between achievement goals and the SWB of students, especially among Chinese students.

Festinger (1954) defined social comparison as a process used by individuals to deliberately select social information to evaluate their opinions and abilities and also to reduce their uncertainty with regard to their own worth. Adolescent students, specifically, are in a unique, critical stage of self-development. During adolescence, the use of social comparisons with peers markedly increases by teachers, parents, and the adolescents themselves, especially in the field of academic achievement (Harter, 2012). Through social comparisons, adolescents seek to affirm their sense of achievement. Although scholastic learning may not be the *only* important activity for adolescents, it is the most highly valued activity among teachers and parents (Wang and Shen, 2009; Zhao, 2013). Therefore, the study of *academic* social comparisons is an important sub-domain of social comparison research for adolescent students in school. Due to differing motivational orientations, adolescent students choose different directions to make academic social comparisons. Butler’s (1992) study found that there were parallel relations between achievement goal orientations and the focus of comparisons. Specifically, mastery goals demonstrate a parallel relation to self-improvement whereas performance goals demonstrate parallel relations to self-evaluation and self-reinforcement, suggesting that achievement goal orientations relate to the direction of academic social comparisons. As Liang’s (2006, unpublished) study has found, the direction of social comparisons may have an effect on SWB as well. Therefore, we hypothesized that the direction of academic social comparisons (i.e., upward vs. downward) may be a key psychological mechanism that accounts for the relations between achievement goal orientations and SWB in school among adolescent students.

### Subjective Well-Being in School

Grounded in Diener’s theory of SWB, Tian (2008) has specifically conceptualized SWB in school as how students subjectively evaluate and emotionally experience their school lives. Furthermore, she has proposed a multidimensional model of SWB in school. In her model, SWB in school consists of students’ reports of school satisfaction (SS) and affect experienced in school. SS refers to a student’s subjective and cognitive evaluations of school life using her or his own standards related to several specific school life domains (e.g., academic learning, teacher-student relationships). Affect in school (AS) refers to a student’s frequency of positive and negative emotions specifically experienced during the school day. Based on this model, Tian et al. (2015a) developed the Brief Adolescents’ Subjective Well-Being in School Scale (BASWBSS), which has shown good psychometric properties in Chinese adolescents.

### Achievement Goal Orientation and Its Relation with Subjective Well-Being in School

An assumption in achievement goal theory is that students have differing goals or differing reasons for engaging or not engaging in learning activities; students also have differing standards for evaluating the outcomes of learning activities (Ames, 1992; Urdan and Schoenfelder, 2006; Patrick et al., 2011). Early goal theory emphasized two goal perspectives: Mastery goals and performance goals (e.g., Ames and Archer, 1988; Nicholls, 1989). Later, Elliot and Church (1997) proposed a trichotomous theory, further dividing the performance goals into approach and avoidance orientations. Therefore, three types of goal orientations were identified: mastery, performance-approach, and performance-avoidance. Students who are focused on developing competence through an emphasis on learning, deepening understanding, and enhancement are thought to be displaying a *mastery goal orientation*. Students with a *performance-approach goal orientation* are focused on the demonstration of competence relative to others by trying to outperform relevant others. Conversely, students with a *performance-avoidance goal orientation* are focused on avoiding looking incompetent and being outperformed by others (Elliot, 2005).

Previous research has shown that achievement goal orientations yield important implications for SWB. For example, Chen and Yu (2009) have found that achievement goal orientations related significantly to students’ SWB. To be specific, students’ focus on mastery orientations is associated with various positive and adaptive patterns of coping and affect (Linnenbrink, 2005; Daniels et al., 2008). With respect to well-being, some studies suggest that performance-approach goals are not maladaptive (Sideridis, 2005) whereas performance-avoidance goals have been linked with maladaptive outcomes, such as hopelessness and shame (Pekrun et al., 2006), stress, anxiety (Smith et al., 2002; Sideridis, 2005). However, there has been little research concerning the effect of achievement goal orientations on domain-specific SWB, such as adolescents’ SWB specifically experienced in the context of their school lives. As noted, community psychologist Sarason (1997) summarized years ago, “Wellness is an individual phenomenon, but it is always embedded in an interpersonal, social-familial, or institutional context” (p. ix). Schools are regarded by adolescents as a major, specific life domain, among others, such as family, community, and peers (Huebner, 1994; Seligson et al., 2003). Hence, it seems worthwhile to explore the relations between adolescents’ achievement goal orientations and SWB in school in particular. Yue (2010) found that mastery goal orientations and performance-approach goal orientations were both positively correlated with SS whereas performance-avoidance goal orientations were negatively linked with SS in high school students. Based on Yue’s findings, we expected that mastery goal orientations and performance-approach goal orientations both had significantly positive correlation with SWB in school whereas performance-avoidance goal orientations had significantly negative correlation with SWB in school among adolescents.

### Academic Social Comparison Direction as a Mediator

Academic social comparison can be defined as a process to obtain information regarding a student’s ability and learning level by comparing his or her academic performance with that of others (Xu, 2005). According to the different ability levels of targets, academic social comparison is typically divided into three directions, including upward academic social comparison, parallel academic social comparison, and downward academic social comparison. Upward academic social comparison involves students’ comparisons with others who exceed their comprehensive learning levels and abilities. Parallel academic social comparisons involve students’ comparisons to others of similar comprehensive learning levels and abilities. Downward academic social comparison involves students’ comparisons with others who display inferior learning levels and abilities (Xu, 2005). Some researchers (Wills, 1981; Collins, 1996) suggest that when individuals compare themselves with others who are different from them, they obtain richer, more differentiated information regarding self-evaluations. Therefore, this study addressed upward academic social comparisons and downward academic social comparisons exclusively.

Several studies have demonstrated that achievement goal orientations relate to social comparisons. Nevertheless, divergent findings have been obtained regarding the nature of the association between the two sets of variables. Specifically, researchers have consistently demonstrated that performance goals are related significantly to social comparison. However, differing findings have been reported concerning the relation between mastery goals and social comparison. On the one hand, Ames (1992) found that mastery goals were uncorrelated with social comparison. On the other hand, other studies have suggested that individuals with mastery goals compare their abilities with others for the purpose of self-improvement (Wood, 1989; Butler, 1995). Taking the unique developmental features of adolescent students into consideration, they typically pay close attention to all aspects of self-development, and they endeavor to show capable and mature selves (Harter, 2012). Furthermore, Régner et al. (2007) demonstrated that mastery goals were positively associated with students’ tendency to engage in social comparison, even after controlling for both performance-approach and performance-avoidance goals. Accordingly, for purposes of self-improvement in academic learning, students with mastery goals would be expected to make academic comparisons. However, there has been little research focusing on the relations between achievement goal orientations and the directions of social comparison. Therefore, in the current study, we further explored the relations between achievement goal orientations and the academic social comparison directions. Referring to the definitions of different types of achievement goal orientations, we anticipated that (1) to acquire more information on self-improvement, students with *mastery goal orientations* would make more upward academic social comparisons; (2) to demonstrate competence and outperform others, students with *performance-approach goal orientations* would also make more upward academic social comparisons; (3) to avoid looking incompetent, students with *performance-avoidance goal orientations* would make more downward academic social comparisons.

Recent research suggests that adolescents’ academic social comparisons influence a variety of important school-related variables. For example, Liu (2010, unpublished) revealed that academic self-efficacy is significantly, positively related to upward academic social comparisons whereas academic self-efficacy is significantly, negatively related to downward academic social comparisons. Wang (2011, unpublished) found that upward academic social comparisons significantly predicted students’ energy, dedication, and attention in learning engagement in school. Based on the above theory and research, we expected that academic social comparison directions should mediate achievement goal orientations and school variables. However, in the few existing studies linking academic social comparison directions to school-related variables, the researchers have focused mainly on achievement-related outcomes, such as learning engagement and self-efficacy, with little attention focused on broader-well outcomes, such as SWB in school. Thus, we addressed whether the direction of academic social comparisons mediates the association between achievement goal orientations (i.e., mastery goals, performance-approach goals and performance-avoidance goals) and adolescents’ SWB in school.

## The Current Study

Taking it into consideration the well-established effect of emotional stability on SWB (Hills and Argyle, 2001; Vittersø, 2001), we controlled emotional stability to examine the relations among adolescent students’ achievement goal orientations, direction of academic social comparisons, and SWB in school. Specifically, we formulated two hypotheses: (1) mastery goal orientations and performance-approach goal orientations will both be positively related to SWB in school whereas performance-avoidance goal orientations will be negatively related to SWB in school among Chinese adolescents; (2) the direction of academic social comparisons will mediate the relations between achievement goal orientations and SWB in school among Chinese adolescent students. More specifically, compared with downward academic social comparisons, upward academic social comparisons will show a stronger mediating effect on the relation between mastery goal orientations and SWB in school, as well as on the association between performance-approach goal orientations and SWB in school; to the contrary, downward academic social comparisons will show a stronger mediating effect on the association between performance-avoidance goal orientations and SWB in school compared to upward academic social comparisons.

## Materials and Methods

### Participants

The convenience sample was drawn from public middle schools in a city located in the province of northern China. A total of 905 students (448 males) completed the questionnaires. Eighteen males and four females were excluded from the analyses due to the missing data on their surveys. The resulting number of valid participants was 430 males and 453 females with response rates of 95.98 and 98.91%, respectively. All participants were from 7th grade and the mean age of the participants was 12.99 years (*SD* = 0.68) with a range of 10–15 years. Almost all of the participants were from middle-income families with parents who earned at least a high school degree.

### Measures

#### Achievement Goal Orientations

Students’ achievement goal orientations were assessed using Achievement Goals Scale (AGS; Jiang and Liu, 2005), which includes three dimensions: Mastery goals, performance-approach goals, and performance-avoidance goals. The items were modified from Elliot and Church’s (1997) AGS. The dimension of mastery goals consists of five items (e.g., “I desire to completely master the material presented in the class.”). The dimension of performance-approach goals consists of six items (e.g., “I am motivated by the thought of outperforming my peers in the class.”). The dimension of performance-avoidance goals consists of five items (e.g., “I worry about the possibility of getting a bad grade in the class.”). Items were rated on a 5-point Likert scale, with response options ranging from 1 (completely disagree) to 6 (completely agree). The AGS has shown adequate support for its reliability and validity in Chinese adolescents (Jiang and Liu, 2005). In this study, Cronbach’s alphas for the subscales were 0.84 for mastery goals, 0.86 for performance-approach goals, and 0.78 for performance-avoidance goals, respectively.

#### Academic Social Comparison Direction

The direction of students’ academic social comparisons was measured using the dimensions of upward academic social comparison and downward academic social comparison from the Academic Social Comparison Direction Subscale (ASCDS; Xu, 2005). The dimension of upward academic social comparison consists of six items (e.g., “I like comparing my academic record with that of my classmates who are better at learning than me”). The dimension of downward academic social comparison consists of six items (e.g., “I like comparing my academic record with that of my classmates who are worse at learning than me”). All items were rated on a 5-point Likert scale, with response options ranging from 1 (completely disagree) to 5 (completely agree). A higher score indicated a higher level on the relevant measure of academic social comparison direction. In this study, Cronbach’s alphas for the subscales were 0.91 for upward academic social comparison and 0.92 for downward academic social comparison.

#### Subjective Well-Being in School

Students’ subjective well-being in school was measured using the BASWBSS (Tian et al., 2015a). The BASWBSS is an 8-item self-report scale comprised of two subscales: SS and AS. The SS subscale consists of six items (e.g., “The teachers’ instructional methods and quality are good.”). Items were rated on a 6-point scale, with response options ranging from 1 (strongly disagree) to 6 (strongly agree). The AS subscale consists of two items. One item assessed the frequency of positive affect (PA) in school, and the other item assessed the frequency of negative affect (NA) in school. The PA item was worded as “In school, the frequency of my pleasant feelings is…”. The NA item was worded as “In school, the frequency of my unpleasant feelings is…”. Both items were rated on a 6-point scale with response options ranging from 1 (never) to 6 (always). The score for the SS subscale was computed by averaging the responses to the six items. The score for the AS subscale was computed by subtracting the NA from the PA score. Finally, the SS and AS subscale scores were summed to create a total BASWBSS score. Empirical support for this SWB in school model and the BASWBSS has been garnered with Chinese adolescents (Tian et al., 2015a). In this study, the Cronbach’s alpha coefficient for the SS subscale was 0.78.

#### Emotional Stability

The personality trait of emotional stability was measured using the Emotional Stability subscale (ESS) of the Abbreviated Junior Eysenck Personality Questionnaire (JEPQR-A; Francis, 1996), which is a widely used measure based on Eysenck’s theory of personality. The ESS consists of six items (e.g., “Do you often feel fed-up?”). Participants responded to questions in a “yes/ no” format, with “yes” scored as 2, and “no” scored as 1. Higher scores thus indicated higher levels of the emotional stability. The convergent validity of the JEPQR-A is supported by high correlations between the JEPQR-A and the longer version, the JEPRA, as reported by Francis (1996). In this study, the Cronbach’s alpha coefficient for the Emotional Stability Scale was 0.65.

### Procedure

The study was approved by the Human Research Ethics Committee of South China Normal University and the relevant school boards, principals, and teachers. Following the approvals, letters describing the study and consent forms were sent to the students’ parents. Only the students who brought back consent forms signed by a parent and who gave their own assent took part in the study. A packet of self-report measures was administered to groups of about 50 students at a time in a regular classroom environment by a trained graduate assistant. The participants all received identical verbal and written instructions from the trained assistant. The students were allowed to take as much time as needed to complete the packet of measures. The graduate assistants were available during survey completion if participants had any questions about the questionnaires. The questionnaires were collected immediately upon completion. After students finished all the measures, they were debriefed regarding the purpose of the investigation. In addition to the formal measures described above, students were asked to provide information about their age and gender.

### Data Analysis

Given our relatively large sample size and small amount of missing data (i.e., 2.49%), the missing data were handled using the listwise deletion procedure, which is acceptable when the loss of cases due to missing data is less than 5% (Graham, 2009). The data analysis plan followed four steps. Firstly, for the initial descriptive analyses and Pearson correlations, the SPSS 17.0 was used. Secondly, confirmatory factor analysis (CFA) using AMOS 17.0 (Arbuckle, 2009) was performed to test the factorial structure of each scale. Thirdly, the two-step procedure recommended by Anderson and Gerbing (1988) was adopted to analyze the mediation effects. The measurement model was first tested to assess the extent to which each of the latent variables was represented by its indicators. If the measurement model was accepted, then the structural model was tested via the maximum likelihood estimation in the AMOS 17.0 program. Structural model fit was analyzed using multiple indicators. First, Chi square (χ^2^) was considered. Traditionally, a good factor structure is inferred when the χ^2^ likelihood ratio is non-significant. However, given the sensitivity of the χ^2^ statistic to sample size (Saris, 1982; Jöreskog and Sörbom, 1996), we supplemented this approach with other goodness-of-fit-measures. These measures were the comparative fit index (CFI), the Incremental Fit index (IFI), the Tucker Lewis index (TLI), and the root mean-square error of approximation (RMSEA) along with its 90% confidence interval (CI; Anderson and Gerbing, 1988; Browne and Cudeck, 1993; Byrne, 2001). For the CFI, IFI, and TLI indices, values greater than 0.90 are typically considered acceptable, and values greater than 0.95 indicate a good fit of the data (Hu and Bentler, 1999; Byrne, 2010). For well-specified models, a RMSEA of 0.06 or less reflects a good fit (Hu and Bentler, 1999; Tabachnick and Fidell, 2007). Finally, bootstrapping, employing 1000 samples, was used for testing significance of the mediated effects and to produce bias-corrected percentile CIs (MacKinnon, 2008). Subsequently, the size of the mediating effect was calculated.

## Results

### Descriptive Statistics

**Table 1** showed that there was no non-normality in the data. Univariate skewness of 2.0 and higher and kurtosis of 7.0 and higher are considered moderate to high non-normality and have been found to create problems in analyses (Muthén and Kaplan, 1992; Curran et al., 1996). However, all variables in the present study demonstrated values well below these levels.

**Table 1:** Descriptive statistics and correlations for the observed variables (*N* = 883).

Variable	*M*	*SD*	Skew	Kurt	1	2	3	4	5	6
1.MG	3.64	0.78	-0.40	0.34	—					
2.PAPG	3.61	0.76	-0.40	0.70	0.62^∗∗^	—				
3.PAVG	3.46	0.80	-0.45	0.39	0.29^∗∗^	0.51^∗∗^	—			
4.UASC	3.47	0.90	-0.62	0.32	0.54^∗∗^	0.48^∗∗^	0.08^∗^	—		
5.DASC	2.40	1.00	0.25	-0.72	-0.15^∗∗^	-0.05	0.11^∗∗^	-0.03	—	
6.SWBS	7.43	2.46	-0.66	0.47	0.30^∗∗^	0.18^∗∗^	-0.04	0.29^∗∗^	-0.15^∗∗^	—
7.ES	1.59	0.28	-0.32	0.75	0.14^∗∗^	-0.02	0.25^∗∗^	0.19^∗∗^	-0.18^∗∗^	0.30^∗∗^

*MG, Mastery Goals; PAPG, Performance-approach Goals; PAVG, Performance-avoidance Goals; UASC, Upward Academic Social Comparison; DASC, Downward Academic Social Comparison; SWBS, Subjective Well-being in School; ES, Emotional Stability. ^∗^p < 0.05; ^∗∗^p < 0.01.*

Preliminary analyses of all study variables, presented in **Table 1**, showed that the correlations among mastery goals, performance-approach goals, performance-avoidance goals and upward academic social comparison were all positive and statistically significant. Mastery goals were significantly negatively related to downward academic social comparison and significantly positively related to SWB in school. Performance-approach goals were significantly positively related to SWB in school but not significantly related to downward academic social comparison. Performance-avoidance goals were positively related to downward academic social comparison but not significantly related to SWB in school. Moreover, upward academic social comparison was significantly positively related to SWB in school whereas downward academic social comparison was significantly negatively related to SWB in school. Also, emotional stability correlated positively with SWB in school.

### The Measurement and Structural Models

We first tested the factorial structure of each scale via CFA. The findings showed that all the scales exhibited acceptable fit indices (**Table 2**) with acceptable factor loadings.

**Table 2:** The confirmatory factor analysis (CFA) results for all scales (*N* = 883).

Scale	χ^2^	*df*	CFI	IFI	TLI	RMSEA (90% CI)
AGS	427.34	101	0.94	0.94	0.93	0.06 (0.055–0.067)
ASCDS	131.82	53	0.99	0.99	0.99	0.04 (0.032–0.050)
BASWBSS	97.92	17	0.96	0.96	0.93	0.06 (0.060–0.088)
ESS	38.94	9	0.95	0.95	0.91	0.06 (0.042–0.082)

*AGS, Achievement Goals Scale; ASCDS, Academic Social Comparison Direction Subscale; BASWBSS, Brief Adolescents’ Subjective Well-Being in School Scale; ESS, Emotional Stability Scale. 90% CI means 90 % confidence interval.*

The measurement model consisted of seven latent factors (mastery goals, performance-approach goals, performance-avoidance goals, upward academic social comparison, downward academic social comparison, SWB in school and emotional stability) and 36 observed variables. A full measurement model was also tested for divergent validity of the latent factors. The results indicated this model provided an acceptable fit to the data: χ2 = 1325, *df* = 579, *p* < 0.001, CFI = 0.95, TLI = 0.94, IFI = 0.95, RMSEA = 0.04 (90% CI of the RMSEA = 0.036–0.041). The measured variables’ loadings on the latent variables were all statistically significant (*p* < 0.001), signifying that the latent variables were adequately measured by their indicators.

**FIGURE 1:**
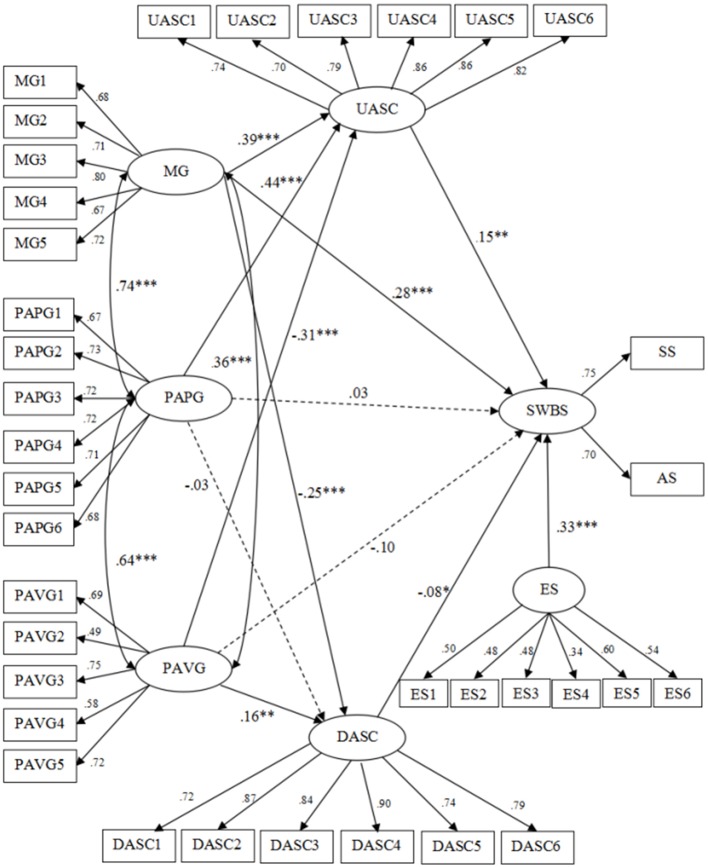
**The Mediation Model (*N* = 883).** MG, Mastery Goals; PAPG, Performance-approach Goals; PAVG, Performance-avoidance Goals; UASC, Upward Academic Social Comparison; DASC, Downward Academic Social Comparison; SWBS, Subjective Well-being In School; ES, Emotional Stability. MG1, Mastery Goals- Composite 1……; PAVG5, Performance-approach Goals- Composite 5; UASC1, Upward Academic Social Comparison- Composite 1……; DASC6, Downward Academic Social Comparison- Composite 6; ES1, Emotional Stability- Composite 1…… ES6, Emotional Stability- Composite 6; SS, School Satisfaction; AS, Affect in School. Solid lines mean the path coefficients are significant while dotted lines mean the path coefficients are not significant. ^∗^*p* < 0.05; ^∗∗^*p* < 0.01; ^∗∗∗^*p* < 0.001.

The structural model included direct paths from achievement goal orientations to SWB in school and two mediators between achievement goal orientations and SWB in school. Results (**Figure 1**) showed that the standardized path coefficients from performance-approach goals to downward social comparison, performance-approach goals to SWB in school, and performance-avoidance goals to SWB in school were non-significant (β = -0.03, *p* > 0.05; β = 0.03, *p* > 0.05; β = -0.10, *p* > 0.05).

### Mediational Effects of Academic Social Comparison Direction

The bootstrap procedure recommended by Shrout and Bolger (2002) was used to examine the significance levels of indirect effects for the hypothesized model. Bootstrapping is a procedure in which a number of samples (e.g., 1, 000) are taken from the original data by random sampling with replacement. The indirect effect in each of these bootstrap samples is computed. We generated 1000 bootstrapping samples from the original data set (*N* = 883) by random sampling.

If the 95% CI for the indirect effect estimate does not include zero, it can be concluded that the indirect effect is statistically significant at the 0.05 level (Shrout and Bolger, 2002). With the exception of one indirect effect (performance approach goals → downward academic social comparison → SWB in school), the results shown in **Table 3** indicated that the 95% CI for the other five indirect effects did not include zero, demonstrating that the other five indirect effects were statistically significant. As shown in **Table 3**, upward social comparison exerted a significant indirect effect on the relations between the three achievement goal orientations and SWB in school. The size of the mediating effect of mastery goals on SWB in school through upward social comparison was 0.06, which accounted for 16.7% of the total effects (0.36). The size of the mediating effect of mastery goals on SWB in school through downward social comparison was 0.02, which accounted for 5.6% of the total effects. The size of the mediating effect of performance-avoidance goals on SWB in school through upward social comparison was -0.05, which accounted for 31.3% of the total effects (-0.16). The size of the mediating effect of performance-avoidance goals on SWB in school through downward social comparison was -0.02, which accounted for 6.3% of the total effects. Furthermore, upward academic social comparison typically mediated the relation between performance-approach goals and SWB in school, and the size of the mediating effect of performance-approach goals on SWB in school through upward social comparison was 0.07, which accounted for 70% of the total effects (0.1).

**Table 3:** Bootstrap analyses of the magnitude and statistical significance of indirect effects.

Independent variable	Mediator variable	Dependent variable	β standardized indirect effect	SE of mean	95% CI mean indirect effect (lower and upper)
MG	UASC	SWBS	0.39^∗^0.15 = 0.06	0.005	0.006, 0.156
	DASC	SWBS	-0.25^∗^(-0.08) = 0.02	0.003	0.0008, 0.067
PAPG	UASC	SWBS	0.44^∗^0.15 = 0.07	0.006	0.006, 0.179
	DASC	SWBS	0.03^∗^(-0.08) = 0.002	0.004	-0.0002, 0.029
PAVG	UASC	SWBS	-0.31^∗^0.15 = -0.05	0.004	-0.059, -0.011
	DASC	SWBS	0.16^∗^(-0.08) = -0.01	0.003	-0.003, -0.0003

*MG, Mastery Goals; PAPG, Performance-approach Goals; PAVG, Performance-avoidance Goals; UASC, Upward Academic Social Comparison; DASC, Downward Academic Social Comparison; SWBS, Subjective Well-being in School.*

## Discussion

We investigated the important roles of the direction of academic social comparisons on the relations between Chinese adolescent students’ achievement goal orientations and SWB in school. The major findings of this study were twofold. First, mastery goal orientations and performance-approach goal orientations both showed statistically significant, positive correlations with SWB in school whereas performance-avoidance goal orientations showed a statistically significant, negative correlation with SWB in school among the Chinese adolescents. Second, upward academic social comparison mediated the relation between the three types of achievement goal orientations (i.e., mastery goals, performance-approach goals and performance-avoidance goals) and SWB in school whereas downward academic social comparison mediated the relation between mastery goal orientations and SWB in school as well as the relation between performance-avoidance goal orientations and SWB in school.

### Achievement Goal Orientations and Subjective Well-Being in School

As hypothesized, mastery goal orientations and performance-approach goal orientations were positively related with SWB in school. Our results are consistent with those of Kaplan and Maehr (1999) who reported that the pursuit of mastery goals was positively associated with general indices of well-being (e.g., positive peer relationships, good impulse control, and positive school-related affect) among sixth grade students. Moreover, several researchers have found a positive relation between performance-approach goals and effort, persistence, and academic achievement (Harackiewicz et al., 2002; Law et al., 2012). Hence, it is reasonable that mastery goal orientations and performance-approach goal orientations are beneficial to SWB in school.

Contrary to expectations, we did not find that performance-avoidance goal orientations were related significantly to SWB in school. However, bivariate correlations among study variables revealed that the correlations among mastery goals, performance-approach goals and performance-avoidance goals were all positive and significant at the *p* < 0.01 level. Because the relation between performance-avoidance goals and SWB in school in the SEM may have been influenced by mastery goals and performance-approach goals, we subsequently controlled for mastery goals and performance-approach goals and found the partial correlation between performance-avoidance goals and SWB in school was -0.159, which was significant at the *p* < 0.01 level. Thus, the performance-avoidance goal orientation was also significantly and negatively related with SWB in school. In conclusion, our results are consistent with those of Zhao and Jin (2008) who showed that mastery goals and performance-approach goals were positively linked to SWB whereas performance-avoidance goals were negatively linked to SWB in college students.

### Mediational Role of Academic Social Comparison Direction

Consistent with our hypothesis (2), upward academic social comparisons mediated the relations between achievement goal orientations (i.e., mastery goals, performance-approach goals and performance-avoidance goals) and SWB in school among Chinese adolescent students. More specifically, students with mastery goal orientations were inclined to make upward academic social comparisons. In line with the definition, mastery goal orientations focus on the improvement of ability. People with mastery goal orientations tend to conceptualize ability as a construct that is malleable and which can be improved through greater effort (Elliot, 1999). Because upward academic social comparisons offer the advantage of providing additional information relevant to how to improve, students with mastery goal orientations tend to prefer making upward academic social comparisons. Furthermore, students with performance-approach goal orientations appeared also more likely to make upward academic social comparisons perhaps because students with performance-approach goal orientations not only strive to improve compared to their own previous levels, but also strive to understand their progress relative to that of other students, in this case students with high abilities. What may be important for such students is to know that they performed better than or at least equal to students of high ability levels. To the contrary, students with performance-avoidance goal orientations rarely made upward academic social comparisons, which is consistent with the definition of performance-avoidance goal orientations in which in students with performance-avoidance goal orientations are concerned primarily with evading appearing incompetent.

With regard to mediating effects of downward academic social comparisons, the results were partially consistent with our expectations. Specifically, downward academic social comparisons mediated the relation between mastery goal orientations and SWB in school as well as the relation between performance-avoidance goal orientations and SWB in school. Students with mastery goal orientations rarely made downward academic social comparisons, again perhaps because they gained little information regarding self-improvement relative to high-achieving peers. To the contrary, students with performance-avoidance goal orientations appeared to prefer making downward academic social comparisons, perhaps in order to evade appearing incompetent and maintain adequate self-worth.

Our study suggested possible important cultural differences in the origins of individual differences in adolescents’ SWB in school. Specifically, regarding the relations between academic social comparisons and SWB in school, Chinese students’ upward academic social comparisons appear to be related to higher SWB in school whereas their downward academic social comparisons appear to be related to lower SWB in school. Upward social comparisons as well as downward social comparisons have two possible effects (i.e., positive or negative effects) on persons’ experiences and moods (Smith, 2000; Buunk et al., 2005). Smith (2000) suggests that the results of comparisons derive from two major factors, that is, whether the comparison is upward or downward and the position that the person takes in relation to the target. Considering the two factors, there are four different possible social comparison processes encompassing upward or downward contrast and upward or downward identification. From the perspective of mechanisms of social comparison, in our study, the students identified with the targets whether they made upward or downward academic social comparisons. In other words, the students made both upward identification and downward identification comparisons. In contrast, some research in western countries revealed that the pattern of social comparison most often observed in school settings involved upward identification combined with downward contrast (Boissicat et al., 2012). These differing results may reflect cultural differences that relate to social comparisons and SWB in school. As previously mentioned, Chinese individuals typically possess a highly context-sensitive self, so that when they choose comparison targets, their self-evaluations are also influenced by the targets. To the contrary, westerners are inclined to make self-judgments based more on their individual thoughts and feelings (Suh, 2007). This possibility is consistent with the study by Ye et al. (2012), which demonstrated that upward comparisons are associated with higher SWB, and downward comparisons are associated with lower SWB in Chinese adolescent students (Mean age = 16.62). Again, additional research is needed to clarify these findings.

### Limitations and Future Direction

This study has some important limitations that should be acknowledged. First, even though a large, heterogeneous sample was obtained, our study was cross-sectional in nature, precluding causal inferences. Because our literature reviewed failed to find any relevant longitudinal studies that addressed the directionality of the relations for any two variables among achievement goal orientations, academic social comparison directions and SWB in school, our study represented an exploratory, first step toward investigating the relations among the variables of interest. However, given our findings, we hope to conduct further longitudinal research that addresses the directionality of the associations among the variables. Second, all data were based on students’ self-reports, which could lead to common method variance issues. The use of multiple methods of assessment would be beneficial in future research. Third, key individual difference factors that may exert an effect on social comparison direction could be explored in future research, like self-esteem and additional personality traits, to more comprehensively understand the development of adolescents’ SWB in school. Finally, contextual factors (e.g., classroom goal structures), which influence adolescent students’ achievement goal orientations, deserve greater attention.

Caution should be also exercised in deriving conclusions from our study given that the effect sizes were variable, with some effects quite small. For example, compared to downward academic social comparisons, upward academic social comparisons demonstrated a stronger mediating effect for Chinese students with mastery goal orientations. Nevertheless, the magnitudes of the mediating effects of both upward and downward comparisons on the association between mastery goals and SWB in school were both small. For another example, upward academic social comparisons demonstrated a stronger mediating effect than downward comparisons for Chinese students with performance-avoidance goal orientations. Furthermore, the mediating effect of upward academic social comparisons on performance-avoidance goals and SWB in school accounted for 31.3% of the total effects whereas that of downward academic social comparisons accounted for only 6.3% of the total effects. It is noteworthy that Chinese students with performance-approach goals more typically made upward academic social comparisons, accounting for 70% of the total effects. Coupled with the cross-sectional nature of the data, the modest effect sizes suggest caution in deriving conclusions from the study.

### Implications

Although this study reflected the above limitations and a need for additional research, the results suggested possible implications related to the links among Chinese adolescent students’ achievement goal orientations, academic social comparison directions, and SWB in school. First, the findings revealed that mastery goal orientations and performance-approach goal orientations were positively related to SWB in school. Thus, school professionals and parents might want to consider promoting Chinese adolescent students’ positive SWB in school through the cultivation of students’ mastery goal orientations and performance-approach goal orientations. Second, the findings suggest that Chinese school professionals might also consider encouraging Chinese adolescent students to make upward academic social comparisons to improve their SWB in school. One plausible explanation for the finding may be that Chinese students identify with their self-selected targets. Furthermore, given that students who share some similarities with upward comparison targets (e.g., sex, age, and possibly other attributes), appear to more easily identify with their comparison targets (see Dijkstra et al., 2008, for a review), educators and parents may seek to guide students to not only make upward academic social comparisons, but to also to choose targets resembling them in an effort to improve their SWB in school. Of course, the above implications are highly tentative in nature and await further research, particularly experimental and longitudinal studies that address the directionality of the linkages.

## Author Contributions

LT, TY, and EH substantially contributed to the conception and the design of the work. LT and TY contributed to the acquisition of data. LT and TY analyzed and interpreted the data. LT prepared the draft and TY and EH reviewed it critically and gave important intellectual content. LT, TY, and EH worked for the final approval of the version to be published. LT, TY, and EH are accountable for all the aspects of the work in ensuring that questions related to the accuracy or integrity of any part of the work are appropriately investigated and resolved.

## Conflict of Interest Statement

The authors declare that the research was conducted in the absence of any commercial or financial relationships that could be construed as a potential conflict of interest.
